# Editorial: Covid-19 special collection

**DOI:** 10.4102/phcfm.v12i1.2466

**Published:** 2020-05-04

**Authors:** Sunanda C. Ray

**Affiliations:** 1 School of Public Health, University of the Witwatersrand, Johannesburg, South Africa; 2 Department of Community Medicine, College of Health Sciences, University of Zimbabwe, Harare, Zimbabwe

It is a well-known story. Disease epidemics by their nature sweep through countries, exposing underlying weaknesses and vulnerabilities, and, in the process, eventually revealing inequalities in access to healthcare and means of protection. Our challenge in African countries with the Coronavirus disease 2019 (Covid-19) pandemic is how to change these threats into opportunities so that we could be better prepared to manage our response to this pandemic, and also to prepare for the next and possibly worse infectious disease pandemic. A colleague with experience from the 2014–2015 Ebola epidemic in West Africa advised us to make the most of the calm before the storm, the period between the early cases and widespread community transmission, to get health staff informed and skilled, to run drills and practical training, to organise health facilities to improve patient flow, to explore strategic approaches that describe clear roles, tasks and communications, and to develop ways of supporting each other when things get worse (Johnson O, 2020, personal communication, 04 April 2020).

The epidemic has evolved from those who could afford to travel and brought the infection from distant lands to local transmission through social, work and other networks. It will initially spread through urban areas and to areas of high population density, and will impact the poor and less privileged the most. The purpose of publishing special reports in the *African Journal of Primary Health Care and Family Medicine* is to share examples of preparedness and lessons already learnt from the Covid-19 pandemic. We would like to know how we can become better in coping with the crisis. Having stopped to take a breath, how can we re-frame the future so that it is more compassionate, more respectful of diversity and inclusive of all sectors of societies?

We need to share ideas on how to maintain critical health services during this emerging crisis, especially in areas where there is lockdown, to avoid longer term consequences. Primary care services play an important role in this regard and must be part of the strategic emergency response. We can learn from previous epidemics in African countries. The decrease in utilisation of healthcare services during the 2014–2015 Ebola outbreak in Sierra Leone was estimated to cause 2800 excess deaths from malaria, human immunodeficiency virus (HIV) and tuberculosis (TB), nearly three quarters as many as the direct Ebola deaths in the country. Declines in utilisation of family planning, antenatal care, facility delivery and postnatal care services were estimated to translate to 3600 additional maternal, neonatal and stillbirth deaths. Mandatory curfews, border closures, disruption of transport and fear of exposure to infection led to a decline in the numbers of people seeking healthcare. Clinic closures and interruption of routine health services such as HIV and TB treatment, childhood vaccinations and maternity care had long-term consequences on the health of communities.^1,2^

Although we tend to think of cholera in terms of outbreaks, it is endemic in 47 countries of sub-Saharan Africa (SSA). Between 2000 and 2015, 83% of 63 658 deaths from cholera reported to the World Health Organization occurred in SSA.^3^ Mapping of incidence of cases during spikes in incidence (outbreaks) allows for identification of hotspots, areas of high-risk populations, which could be targeted for intensive interventions, to control outbreaks and prevent further transmission.^3,4^ The approach to hotspots could be used in Covid-19 outbreak control also and is underway in South Africa at present, using community health workers to screen people in households for symptoms. An intervention common to both diseases is the promotion of handwashing; therefore, ensuring that soap and water are provided to communities is essential. The second best strategy, for those who are unable to get to a facility to wash their hands, is that hand sanitiser with at least 60% alcohol also needs to be provided. The most vulnerable to both diseases are those who live in overcrowded accommodation, sharing toilets with large numbers. During cholera outbreaks in Zambia and Zimbabwe, donations of soap and water supplies were often provided by civil society organisations, religious institutions and non-governmental organisations (NGOs), along with health education on the importance of handwashing. Engagement of such organisations is essential for Covid-19 also. During the response to the devastation caused by Cyclone Idai, civil society and community organisations coordinated the response in an efficient and timely manner. They provided transport, collected materials needed, identified those who needed support the most and distributed food packages, medications and equipment. In high-income countries, volunteer support for health staff has been notable, including providing emotional and material support for critical care workers.

A targeted hotspot approach may work as an alternative to total lockdown once community transmission has become established but relies on very good information systems linking laboratories with action groups and rapid response teams, transparency of data on case-finding and contact tracing. When vaccinations became available for cholera, identifying hotspots was crucial as a way of targeting areas of greatest ability to benefit.^4^ Inevitably there will not be sufficient vaccine supplies for Covid-19 when they are cleared for general use. Targeting hotspots will ensure that Covid-19 vaccines are used for the most vulnerable and those with best ability to benefit rather than general dissemination.

This is a pandemic that has been unfolding rapidly with the Internet buzzing with new information, sharing protocols, guidelines, research articles, ideas and examples, as well as some fake news. We can learn from the experiences of those who faced these challenges before us. Diaspora doctors in Britain and the United States of America are sharing their workplace experiences in critical care with their counterparts in district hospitals in Zimbabwe. Non-medical journals have carried stories about whether giving oxygen only is as good as putting patients on ventilators. Some of us have learnt for the first time about ‘proning’ and the benefits of this for patients in respiratory distress. The preoccupation with critical care has been a natural progression in understanding why the aim of prevention is to keep total numbers down so that we have fewer seriously sick patients in hospitals needing critical care (5% of those infected). Otherwise we would be fine with having a mild infection with a few days of illness. Sadly, about 1% – 4% of those who get this infection will die from it. These will not all be elderly or those with comorbidities.

Lockdown is easier for people who live in houses with big gardens. It is harder for those who live in crowded homes, sharing their living spaces and toilets with others. If they are not at work or at the shops, they will be sitting outside, rarely alone. There has been frustration with people who seem to ignore the calls to stay at home and for social distancing, as though they do not appreciate the seriousness of this disease. The sad truth is that for many in our communities, they know that if they or their family members get seriously ill, they will die. Even before Covid-19, going into hospital with a serious problem, getting critical care was out of reach and unaffordable. Some have accessed funding for medications from family members abroad through remittances. The majority do the best they can. So Covid-19 is no different for them. They need to earn money through vending or small jobs, and then to join the queue to buy basics for the day to feed the family. They are very aware that they are more likely to die of high blood pressure or diabetes than Covid-19. Some feel they are more likely to die of hunger. These needs are much more real to them than talk about ventilators and oxygen concentrators. Most governments do not pay social benefits or provide food parcels, unless campaigning for an election. Most governments do not consult communities on how they would like to respond to crises like Covid-19.

There are stories of initiatives and innovations that groups all over Africa are exploring in how to transform this crisis into something radical, sustainable and will endure. We must share them and learn from them.
